# The Promise of Intranasal Oxytocin in Treating Borderline Personality Disorder: A Narrative Review

**DOI:** 10.3390/brainsci15070708

**Published:** 2025-06-30

**Authors:** Eleni Giannoulis, Christos Nousis, Lydia-Angeliki Eytaxia, Olga Kaimakami, Ioannis Malogiannis

**Affiliations:** 1 Laboratory of Psychometrics and Neuropsychology, Eginition Hospital, Medical School of Athens, 11528 Athens, Greece; xristosnousis@hotmail.com (C.N.);; 2 Specific Sector of Personality Disorders, Eginition Hospital, Medical School of Athens, 11528 Athens, Greece; ioannis.malogiannis@gmail.com

**Keywords:** BPD, intranasal oxytocin, emotional regulation, social cognition, neuroplasticity, psychotherapy integration

## Abstract

Background/Objectives: Borderline personality disorder (BPD) is a complex psychiatric condition marked by emotional dysregulation, interpersonal instability, and impulsivity. Despite the advances in psychotherapy and pharmacotherapy, many patients show a partial or unstable response. Recent research suggests that oxytocin, a neuropeptide involved in social cognition and emotional regulation, may offer novel therapeutic avenues. Methods: We systematically synthesize evidence from PubMed, PsycINFO, Web of Science, and Google Scholar on oxytocin’s role in BPD, prioritizing studies on neurobiology, emotion regulation, clinical interventions, and adjunctive therapy models. Thirty studies were included and critically appraised using PRISMA and Cochrane’s tools. Due to methodological heterogeneity, no meta-analysis was conducted; instead, the findings were integrated through a narrative synthesis approach. Results: Evidence supports oxytocin’s modulatory effects on amygdala reactivity, prefrontal–limbic connectivity, and hypothalamic–pituitary–adrenal axis function. Intranasal oxytocin appears beneficial for emotional regulation and interpersonal sensitivity, particularly in individuals with early trauma. The reported effect sizes ranged from small (Cohen’s d ≈ 0.40) to large (d ≈ 0.83), though some trials reported null or adverse effects, such as increased hypermentalization. Heterogeneous responses were influenced by factors such as sex, trauma history, and OXTR gene variants. Conclusions: Although intranasal oxytocin shows promise in modulating core neurobiological systems implicated in BPD and enhancing emotion regulation and social cognition, its clinical effects remain variable and context-dependent. The evidence supports cautious exploration of oxytocin as an adjunct to psychotherapeutic interventions rather than as a standalone treatment. Future research should focus on biomarker-informed, stratified trials that account for trauma history, genetic variation, and sex differences to clarify its therapeutic potential.

## 1. Introduction

Borderline personality disorder (BPD) is a severe and common psychiatric condition, with an estimated lifetime prevalence of around 1–2% in the general population and up to 20% among psychiatric inpatients [1,2]. BPD is characterized by pervasive instability in affect regulation, self-image, interpersonal relationships, and impulse control. These core features lead to significant impairment in areas such as interpersonal relationships, academic or occupational performance, and emotion regulation [3,4].

Research consistently shows that BPD disproportionately affects women, with clinical samples often comprising 70–75% female patients [2,5]. However, this gender distribution may partly be due to diagnostic biases and under-recognition in males, who more frequently exhibit externalizing behaviours or comorbidities such as substance abuse [6]. Importantly, BPD can manifest differently across genders: women often present with internalizing symptoms (e.g., affective instability and self-harm), while men may exhibit more externalizing traits (e.g., aggression and antisocial behaviours) [7].

While the prognosis of BPD was historically viewed as poor, it is more nuanced today. Longitudinal studies suggest that symptom remission is possible, particularly for impulsive and affective symptoms, though interpersonal dysfunction and identity disturbances often persist [8]. However, comorbid conditions, including major depressive disorder, PTSD, substance use disorders, and eating disorders, complicate treatment and contribute to increased morbidity and suicidality. Up to 10% of people with BPD die by suicide [2,9].

In light of these challenges, psychotherapy, particularly Dialectical Behaviour Therapy (DBT) and Mentalization-Based Treatment (MBT), is crucial [10,11]. Nevertheless, a significant proportion of patients continue to experience refractory symptoms, indicating the necessity for adjunctive biological interventions.

Emerging research has highlighted the potential role of oxytocin, a neuropeptide centrally involved in social bonding, emotional regulation, and stress modulation, in the pathophysiology and treatment of BPD. Altered oxytocinergic functioning, partly modulated by genetic polymorphisms in the oxytocin receptor gene (OXTR), has been linked to interpersonal hypersensitivity, emotional lability, and impaired attachment, all of which are hallmark features of BPD [12,13,14,15,16]. This has sparked growing interest in the therapeutic use of intranasal oxytocin as an adjunctive treatment, particularly for individuals with a history of early trauma and disrupted attachment patterns. Intranasal administration is considered an effective delivery route because it allows oxytocin to bypass the blood–brain barrier via the olfactory and trigeminal pathways, enabling direct access to the central nervous system structures involved in emotion and social behaviour [17].

In this study, our aim was to systematically examine the current evidence on the role of oxytocin in BPD, focusing on its neurobiological mechanisms, clinical effects, and potential for integration with established psychotherapeutic modalities. By synthesizing findings from neuroscience, psychopharmacology, and psychotherapy research, this review will evaluate the promise and limitations of oxytocin-based interventions for individuals with BPD.

## 2. Materials and Methods

**Figure 1:**
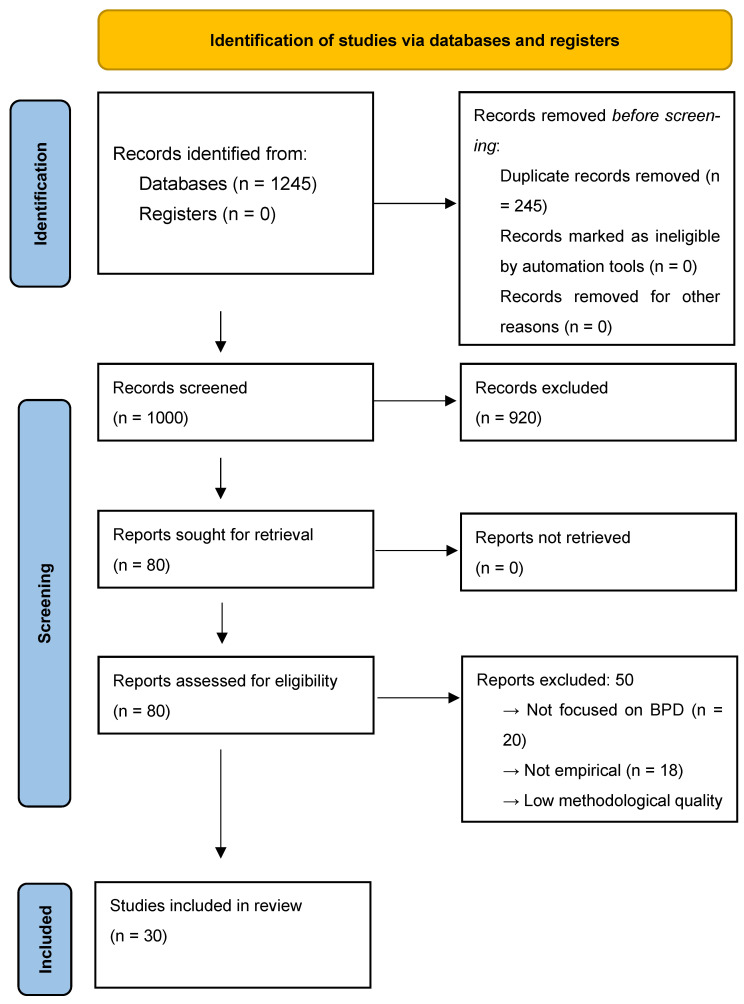
PRISMA flow diagram (Page MJ et al., 2021) [19] illustrating the study selection process. A total of 1245 records were identified through database searches. After removing duplicates, 1000 titles and abstracts were screened. Of the 80 full-text articles assessed for eligibility, 50 were excluded for the following reasons: not focused on BPD (n = 20), not empirical (n = 18), or low methodological quality (n = 12). Thirty studies were included in the final review.

This systematic narrative review was conducted in accordance with the PRISMA 2020 guidelines [18] and aimed to summarize the latest research on the neurobiological and clinical effects of oxytocin, both endogenous and intranasal, in individuals with BPD. Eligible studies included randomized controlled trials, observational studies, neuroimaging investigations, and systematic reviews. The review protocol involved clearly defined inclusion and exclusion criteria, a structured search strategy, dual-reviewer screening and data extraction, and a critical appraisal of study quality. Although 30 studies were included, a meta-analysis could not be performed due to substantial methodological heterogeneity across the studies. The variability in sample characteristics (e.g., gender, trauma history), oxytocin administration protocols (e.g., dosage, timing), outcome measures (e.g., amygdala reactivity, cortisol levels, interpersonal trust), and study designs (e.g., randomized controlled trials (RCTs) vs. cross-sectional studies) prevented meaningful statistical aggregation. Therefore, a narrative synthesis approach was adopted to allow for qualitative integration and an exploration of moderator variables. The study selection process is illustrated in the PRISMA flow diagram (Figure 1).

### 2.1. Literature Search Strategy

A comprehensive search of the peer-reviewed literature was conducted using PubMed, PsycINFO, Web of Science, and Google Scholar between January 2003 and January 2024. The following Boolean logic was used in various combinations:

(“Borderline Personality Disorder” OR “BPD”) AND (“oxytocin” OR “intranasal oxytocin”) AND (“emotion regulation” OR “social cognition” OR “amygdala” OR “HPA axis” OR “therapy”).

The search results were limited to English-language publications involving human subjects. The reference lists of relevant papers were also manually screened to identify additional studies.

### 2.2. Eligibility Criteria

Studies were considered eligible for inclusion if they met the following criteria:

Inclusion criteria:

- Empirical investigations, randomized controlled trials (RCTs), observational studies, systematic reviews, or meta-analyses.
- Focused on oxytocin’s neurobiological mechanisms or the clinical effects of oxytocin in BPD populations.
- Examined either the endogenous or intranasal administration of oxytocin.
- Investigated oxytocin in the context of psychotherapeutic augmentation (e.g., DBT, MBT).

Exclusion criteria:

- Case reports, opinion pieces, editorials, or theoretical essays without empirical data.
- Studies unrelated to BPD or not focused on oxytocin.
- Articles without peer-review validation.

The search included studies published between January 2000 and May 2025, based on the increasing volume of oxytocin-related BPD research during this period.

### 2.3. Study Selection Process

The screening and selection process was carried out independently by two reviewers. First, all titles and abstracts were initially screened based on the predefined eligibility criteria. Full-text articles were then assessed for inclusion. Any discrepancies between the reviewers were resolved through discussion and consensus, and a third reviewer was consulted when necessary. This dual-review procedure was employed at both the screening and data extraction stages to ensure methodological transparency and minimize bias.

The rationale for excluding full-text articles is detailed in the PRISMA flow diagram (Figure 1), which provides a breakdown of exclusions and the reasons for them. This ensures transparency in the study selection process and adherence to systematic review standards.

### 2.4. Data Extraction

Two independent reviewers used a standardized data extraction form to collect information from each eligible study. Extracted variables included:

- Study design (e.g., randomized controlled trial, observational, neuroimaging study).
- Sample characteristics (e.g., size, sex distribution, trauma history).
- Method and dosage of oxytocin administration (e.g., intranasal or endogenous measurement).
- Targeted outcomes (e.g., amygdala activity, prefrontal connectivity, cortisol levels, emotional regulation, and social cognition).
- Moderator variables (e.g., OXTR gene polymorphisms, psychiatric comorbidities).
- Key findings and, where reported, statistical effect sizes (e.g., Cohen’s d).

Any discrepancies between reviewers during data extraction were resolved through discussion, with a third reviewer consulted when needed. The extracted data were synthesized into summary tables (see Appendix A), and thematic categories were used to organize the findings into mechanistic versus clinical domains.

### 2.5. Quality Assessment

The quality of the study was evaluated independently by two reviewers. For randomized controlled trials (RCTs), the Cochrane Risk of Bias Tool was used to assess selection, performance, detection, attrition, and reporting bias. Systematic reviews were assessed using the PRISMA 2020 checklist. Observational studies were evaluated using predefined criteria, including the clarity of the inclusion criteria, the validity of the exposure and outcome measurements, the control of confounding factors, and the appropriateness of the statistical analysis. All disagreements were resolved through consensus. A summary of the methodological quality scores is provided in Appendix A.

Due to the heterogeneity in study design, populations, and outcome measures, a narrative synthesis approach was adopted. Methodological heterogeneity was qualitatively evaluated by comparing design features, intervention protocols, and analytic strategies across studies. These comparisons are reflected in the Results and Discussion Sections.

### 2.6. Data Synthesis

To enhance clarity and address the dual nature of the literature, the results were organized into two primary thematic domains: The first domain comprised studies examining the neurobiological mechanisms of oxytocin relevant to BPD such as amygdala modulation and HPA axis regulation. The second domain comprised studies investigating the clinical effects of oxytocin administration such as symptom changes, therapy outcomes, and adverse effects). This separation enabled for a more targeted synthesis of findings across experimental and therapeutic domains.

However, due to the methodological heterogeneity of the studies in terms of design, outcome measures, populations, and intervention protocols, a quantitative meta-analysis was not feasible. Instead, findings were integrated using a thematic synthesis approach. Neurobiological studies were grouped and analyzed by shared mechanisms (e.g., amygdala modulation, prefrontal–limbic connectivity, HPA axis activity), while clinical trials were synthesized through narrative integration, paying attention to treatment efficacy, adverse effects, and reported effect sizes. Divergent findings were contextualized based on identified moderator variables, such as participant sex, trauma history, and oxytocin receptor gene (OXTR) polymorphisms. This structured approach enabled a multidimensional understanding of oxytocin’s role in BPD to be achieved.

### 2.7. Risk of Bias Assessment

The risk of bias was assessed independently by two reviewers. For randomized controlled trials (RCTs), the Cochrane Risk of Bias Tool (RoB 2) was used to evaluate domains such as randomisation, blinding, outcome measurement, and selective reporting.

For observational studies, a structured quality checklist was used, adapted from NIH’s Quality Assessment Tool for Observational Cohort and Cross-Sectional Studies, to assess the following:

- Clarity of inclusion criteria.
- Valid exposure/outcome measurement.
- Identification and control of confounders.
- Appropriateness of statistical analysis.
- Transparency in reporting.

Studies were rated as having a low, moderate, or high risk of bias, and a summary table of the scores is included in Appendix A.

This review was not pre-registered in PROSPERO or any other international registry, which may limit transparency and reproducibility.

## 3. Results

Overall, the methodological quality of the included studies was moderate to high. Of the twelve randomized controlled trials (RCTs), eight were rated as low risk of bias, while four were classified as having some concerns due to inadequate blinding or unclear randomisation protocols. The quality of the ten observational studies varied; most clearly defined their sample and exposure but had limitations in terms of controlling confounding factors. The three included systematic reviews met the core PRISMA reporting standards, although only one provided a complete risk of bias analysis.

### 3.1. Overview of Study Selection and Characteristics

Out of the 1245 records identified through database searches, 1000 were screened after duplicates were removed. Eighty full-text articles were evaluated for eligibility and fifty were excluded for the following reasons:

- Not focused on BPD (n = 20).
- Theoretical or opinion based without empirical data (n = 18).
- Methodologically inadequate (e.g., no control group or poor outcome reporting) (n = 12).

A total of thirty studies were included in the final review:

- Twelve RCTs.
- Ten observational studies.
- Five neuroimaging studies.
- Three systematic reviews.

Table A1 (Appendix A) summarizes the design characteristics (sample size, oxytocin dosage, and outcome domains) and key findings of the 30 studies included in this review.

The findings are presented in three structured subsections:

(1) Neurobiological mechanisms of oxytocin in BPD;
(2) Effects on emotional regulation and social cognition;
(3) Clinical trials evaluating treatment efficacy and therapeutic integration.

### 3.2. Neurobiological Mechanisms of Oxytocin in BPD

Oxytocin is a neuropeptide produced in the hypothalamus that is subsequently released into the bloodstream by the posterior pituitary gland. It plays a vital role in facilitating social bonding, regulating emotions, and modulating stress responses. The oxytocinergic system interacts with other neurotransmitter systems, including those involving dopamine and serotonin. These neurotransmitters have all been associated with BPD [18,20,21,22,23,24,25].

### 3.3. Oxytocin and Emotional/Social Processing in BPD

In this review, we use the following distinctions to support conceptual clarity. Emotional processing refers to how individuals identify, experience, and regulate their own emotional states. Social cognition pertains to interpreting others’ emotions, intentions, and social cues—often through mechanisms like empathy, theory of mind, and trust evaluation. Interpersonal sensitivity describes the heightened reactivity to social feedback, especially perceived rejection or threat, which is commonly elevated in individuals with BPD. While these constructs are interrelated and often co-occur, maintaining terminologi-cal clarity is essential when evaluating oxytocin’s modulatory effects.

One of the key features of BPD is emotional dysregulation, which often manifests as intense mood fluctuations and heightened sensitivity to relational stressors [23,26,27]. There is evidence to suggest that oxytocin influences emotional processing by acting on the key brain regions involved in emotion regulation, such as the amygdala, prefrontal cortex, and anterior cingulate cortex [18,22,28,29]. Studies have shown that people with BPD have increased amygdala activity, leading to stronger emotional responses to negative stimuli [30,31,32,33,34]. Studies have shown that oxytocin can reduce amygdala activity, enhancing emotional control over emotions in patients with BPD [23,32,34,35,36,37].

Oxytocin also promotes increased activity in the prefrontal cortex, thereby enhancing the cognitive regulation of impulsive emotional reactions [18,35,38]. Heightened connectivity between the limbic system and the prefrontal cortex may improve control over distressing emotions, resulting in a reduction in the frequency and intensity of emotional outbursts described in patients with BPD [18,32,39,40,41,42].

### 3.4. Oxytocin and Its Involvement in Social Cognition

Oxytocin plays a critical role in social cognition, influencing processes such as emotional recognition, trust, empathy, and the interpretation of social cues—domains frequently impaired in individuals with BPD. BPD is also commonly associated with impaired social cognition. This can manifest as hypersensitivity to perceived social rejection, difficulty with decoding social cues, and unstable interpersonal relationships [43,44,45,46,47,48,49]. Oxytocin has also been identified as a promoter of social cognition, operating by increasing the salience of positive social cues and decreasing the detection of threatening stimuli [49,50,51,52,53,54]. This can minimize rejection sensitivity and paranoia, which are prevalent interpersonal difficulties associated with BPD [35,55,56]. However, individuals’ responses to oxytocin can vary considerably. Various studies demonstrate that, while oxytocin may enhance prosociality in some patients with BPD, it can exacerbate symptoms of hypermentalization and mistrust in patients who have experienced severe attachment trauma [23,35,36,41,50,57,58,59]. This suggests that the impact of oxytocin could depend on underlying neurobiological and psychological factors, heightening the need for tailored oxytocin-based interventions [23,60,61].

### 3.5. Clinical Effectiveness of Intranasal Oxytocin

#### 3.5.1. Oxytocin and Depressive Symptoms

Importantly, there is no strong link between oxytocin concentrations and depressive symptoms. This suggests that oxytocin dysregulation is specifically related to BPD rather than general mood disorders [62]. This suggests that therapies targeting oxytocin may selectively treat social cognition and emotional dysregulation in BPD rather than general affective disturbance.

#### 3.5.2. Oxytocin and Stress Response

The hypothalamic–pituitary–adrenal (HPA) axis is often dysregulated in patients with BPD [7,63,64,65,66]. Hyperactive stress responses and elevated cortisol levels are responsible for emotional lability and impulsivity in patients with BPD [2,52,67,68,69]. Evidence indicates that oxytocin modulates HPA axis activity by decreasing cortisol secretion and, consequently, reducing physiological stress levels [34,69,70,71,72,73,74].

The stress-buffering effect could be particularly beneficial for individuals diagnosed with BPD, since heightened stress sensitivity can exacerbate maladaptive coping mechanisms such as self-harm and dissociative behaviours [23,29,36,48,50,59,75,76]. By mitigating the physiological effects of stress, oxytocin can bolster resilience and facilitate healthier emotional regulation processes [73,77,78].

#### 3.5.3. Neuroplasticity and Long-Term Effects

Current evidence suggests that oxytocin plays a role in neuroplasticity by enabling changes in the brain regions involved in emotional and social processing [79,80,81,82,83]. Long-term oxytocin treatment has also been associated with enhanced synaptic plasticity in the amygdala and prefrontal cortex, which may result in long-lasting improvements in emotional regulation and social cognition [80,84,85,86,87,88,89].

The present research suggests that oxytocin can facilitate long-term neural adaptation that enhances chronic emotional and social functioning, as well as short-term symptom alleviation [78,89,90,91,92]. Nevertheless, more research is required to determine the optimal dosage and frequency of oxytocin to achieve long-term therapeutic benefits in patients with BPD [23,50,58,59,60,61,92,93].

#### 3.5.4. Clinical Evidence on Oxytocin Treatment of BPD

*Positive Results*

Several randomized controlled trials (RCTs) have investigated the efficacy of intranasal oxytocin as a treatment for BPD [23,59,60,61,62,64,94]. Bertsch et al. (2013) found that oxytocin treatment reduced social threat hypersensitivity and improved emotional regulation in females with BPD [35]. This concurs with the findings of Domes et al. (2007), who demonstrated that amygdala reactivity decreased following oxytocin treatment, leading to improved emotional processing [29]. Brüne et al. (2013) demonstrated improved interpersonal social cognition and trust-based behaviour, with subjects showing greater cooperation and prosocial behaviour following treatment [57]. Furthermore, studies have shown that oxytocin can reduce hyperreactivity to perceived social rejection, which is a key issue for patients with BPD [35]. Intranasal oxytocin improves symptom severity in patients with major depressive disorder (MDD) lacking BPD, but has no effect on patients with comorbid BPD, revealing that its action varies based on patient characteristics [94].

*Contradictory Results and Weaknesses*

Despite such positive results, the studies show contradictory findings. Some studies have suggested that oxytocin administration can exacerbate hypermentalization, causing individuals to misinterpret social cues and experience increased paranoia [23,41,76,95,96]. However, most research focuses primarily on female subjects, so it is unclear whether these findings can be generalized to male patients with BPD [5,7,97]. Most research examines the acute effects of oxytocin; its long-term effectiveness and potential side effects remain largely unexplored [98,99,100]. Furthermore, environmental influences and genetic vulnerabilities, including polymorphisms in the OXTR gene and trauma in childhood, can influence therapy outcomes, contributing to considerable heterogeneity in the response rates [12,14,52,101].

Most studies have primarily included female participants, which restricts the knowledge regarding oxytocin’s involvement in male BPD populations [62,97]. Furthermore, small sample sizes and variable methodologies restrict the ability to extrapolate the findings [23]. Most studies assess peripheral oxytocin levels (in plasma or serum), which may not reflect central oxytocin activity in the brain [62]. Additionally, the absence of non-invasive real-time oxytocin measurement during social interactions or stress provocation hinders our understanding of dynamic regulation [97]. Most research is cross-sectional, ruling out the causal inferences about whether oxytocin dysregulation is a cause or consequence of BPD [23].

### 3.6. Integration with Psychotherapy: Adjunctive Applications

Due to the complex nature of oxytocin’s actions, it is most effective when used in combination with other treatments [102,103,104]. It may increase emotional regulation capacity by reducing amygdala-mediated reactivity, thereby enhancing engagement in Dialectical Behaviour Therapy (DBT) interventions [10,37]. By improving social cognition, oxytocin could help patients with BPD to better interpret social cues and reduce misunderstandings of others’ intentions [11,48,60,105]. As childhood trauma is a significant factor in the development of BPD, some researchers have suggested combining oxytocin with trauma-informed psychotherapies, such as EMDR or narrative exposure therapy, to enhance emotional processing and therapeutic engagement [7,106,107,108]. While these studies do not report on clinical trials that combine oxytocin directly with psychotherapeutic interventions, they provide a conceptual rationale in support of such integrative approaches.

## 4. Discussion

### 4.1. Summary of Key Findings

This systematic review synthesised the results of 30 empirical studies examining the role of oxytocin in people with BPD. The results were organised into three categories: neurobiological mechanisms, psychological effects on emotional and social processing, and clinical treatment outcomes. Mechanistic studies consistently demonstrated that oxytocin modulates the key neural systems implicated in BPD, primarily by reducing amygdala hyperactivity, enhancing prefrontal–limbic connectivity, and decreasing the activity of the hypothalamic–pituitary–adrenal (HPA) axis. In terms of psychological function, oxytocin was associated with improvements in emotional regulation and social cognition, including reductions in rejection sensitivity and the enhanced interpretation of social functioning. However, these effects were moderated by trauma history and attachment patterns. Clinical trials produced mixed results: while some studies reported an improvement in symptoms, others identified adverse outcomes such as heightened hypermentalization or a lack of therapeutic benefit, particularly in participants with unresolved trauma. Thematic synthesis enabled a structured comparison of the underlying mechanisms, while narrative integration highlighted the differences in clinical outcomes and helped identify patient-specific factors contributing to the variability in therapeutic response. Overall, the evidence indicates that oxytocin’s therapeutic effects are highly context-dependent, modulated by individual-level factors such as sex, developmental adversity, and genetic predispositions. This underscores the heterogeneity of treatment response in BPD. The following discussion is grounded exclusively in the findings synthesized from the included studies, with the clinical and theoretical implications being carefully tied to the reviewed evidence.

### 4.2. Implications of Neurobiological Findings

The neurobiological studies reviewed highlight the regulatory influence of oxytocin on the brain regions and circuits that are central to BPD pathology. Most consistently, oxytocin was found to reduce amygdala reactivity to social and emotional stimuli, which may be a mechanism underlying the reductions in emotional hypersensitivity—a core symptom of BPD. Additionally, oxytocin enhances the functional connectivity between the prefrontal cortex and limbic structures, thereby supporting the modulation of emotional responses from above. This increased integration may encourage the adaption of more adaptive emotion regulation strategies and reduce impulsive behaviours. Oxytocin also appears to buffer stress responses by modulating the HPA axis, thereby contributing to physiological calmness and improved coping in situations of social threat. Taken together, these findings suggest that oxytocin has potential as a neuromodulatory agent, capable of restoring balance in dysregulated affective circuits. However, variation in individual neurobiological profiles, such as baseline oxytocin levels, receptor density, and prior trauma exposure, likely shapes the magnitude and direction of oxytocin’s effects. These results highlight the importance of developing intervention strategies guided by biomarkers and neuroimaging-informed clinical trials to improve the prediction of therapeutic responses in BPD populations.

### 4.3. Translational Value: Emotion Regulation and Social Cognition

Given the interpersonal sensitivity and affective lability that define BPD, oxytocin’s effects on emotional regulation and social cognition are especially relevant. Several studies have reported that oxytocin administration improves emotion recognition, reduces fear responses, and enhances trust in social contexts—effects that may promote more stable interpersonal functioning. Notably, oxytocin appeared to decrease sensitivity to rejection and increase attunement to positive social cues, which could counteract the maladaptive interpersonal schemas commonly experienced by people with BPD. However, these benefits were not uniform. In individuals with histories of attachment trauma or unresolved interpersonal mistrust, oxytocin sometimes exacerbated hypermentalization or suspicion. This suggests that the peptide’s prosocial effects may depend on developmental and relational factors. This bidirectional effect highlights the contextual and person-specific action, emphasizing the importance of tailoring interventions to patients’ trauma histories and relational patterns. These findings point to a promising, yet nuanced, translational pathway: while oxytocin can facilitate improved emotional and social processing in many individuals with BPD, its effectiveness may depend on concurrent psychotherapeutic support and careful patient selection.

### 4.4. Clinical Applications and Integration into Psychotherapy

Evidence from randomized controlled trials assessing the therapeutic utility of intranasal oxytocin for BPD presents a complex picture. While several studies report improvements in emotion regulation, interpersonal trust, and symptom reduction, others fail to show significant benefits or even suggest adverse effects such as heightened paranoia and emotional overinterpretation. These discrepancies may be attributed to differences in sample composition (e.g., predominantly female participants), dosing protocols, trauma exposure levels, and genetic variability (e.g., OXTR polymorphisms).

Importantly, oxytocin may be most effective when used as an adjunct to established psychotherapeutic treatments rather than as a standalone intervention. For example, its capacity to reduce amygdala-mediated fear responses and increase trust may facilitate engagement in Dialectical Behavior Therapy (DBT), especially during emotionally challenging phases. Similarly, in Mentalization-Based Therapy (MBT), oxytocin may help improve patients’ ability to accurately infer others’ mental states and reduce hypermentalization—particularly when administered prior to sessions involving interpersonal triggers.

Moreover, its stress-buffering properties make oxytocin a potential complement to trauma-informed therapies such as Eye Movement Desensitization and Reprocessing (EMDR) or narrative exposure therapy. However, these applications remain largely theoretical; no published trials to date have directly tested oxytocin administration in combination with manualized psychotherapeutic interventions.

To optimize clinical translation, future studies should explore combined treatment models with clearly defined protocols, such as the timing of oxytocin administration relative to therapy sessions and personalized stratification based on biomarkers or trauma history.

### 4.5. Limitations

While this review identified several promising findings, the original studies reviewed exhibited a number of methodological limitations. The majority of studies involved small sample sizes, which reduces statistical power and limits the generalizability of the results. Additionally, there was a marked gender bias, as most samples consisted predominantly of female participants, restricting the applicability of findings to male or gender-diverse individuals with BPD. The literature also displayed considerable design heterogeneity, with variability in study protocols, oxytocin dosages, administration schedules, and outcome measures, all of which hinder meaningful cross-study comparisons or meta-analytic synthesis. A further concern is the lack of long-term data, as most studies assessed only the acute or short-term effects of oxytocin, offering limited insight into its sustained impact on symptomatology or functional outcomes. Measurement concerns also arise from the use of peripheral oxytocin assays, which may not accurately reflect central nervous system levels or activity. Finally, limited control of confounding factors—such as early trauma, comorbid psychiatric conditions, and genetic variability (e.g., OXTR polymorphisms)—was evident in several observational designs, potentially influencing outcome interpretations. These limitations underscore the need for more robust, standardized, and stratified research to clarify oxytocin’s therapeutic potential in BPD. These limitations highlight the need for larger, more rigorously designed trials that account for individual variability in trauma exposure, neurobiology, and genetic susceptibility.

Several limitations of the present review should be acknowledged. First, although the review followed systematic procedures aligned with PRISMA guidelines, it was not pre-registered in PROSPERO or other international registries, which may affect transparency and reproducibility. Second, while a comprehensive search strategy was employed across multiple databases, the review was limited to English-language publications, raising the possibility of language bias and the exclusion of relevant non-English studies. Third, due to the heterogeneity of the methodologies and outcome measures, a meta-analytic synthesis was not feasible, which may limit the precision and quantifiability of the conclusions. Fourth, although the quality of included studies was assessed, the subjective nature of narrative synthesis may introduce interpretative bias, despite efforts to standardize the extraction and integration procedures. Finally, while every effort was made to identify and account for moderating variables (e.g., sex, trauma history, genetic differences), this review remains constrained by the reporting practices of the primary studies, which were not always sufficiently detailed. These limitations suggest caution in overgeneralizing the findings and highlight the need for ongoing refinement in future systematic reviews of oxytocin’s role in BPD. While the findings suggest potential clinical applications for oxytocin in BPD, the conclusions must remain within the scope of the evidence synthesized and await confirmation through further empirical study.

### 4.6. Future Directions and Research Recommendations

The authors emphasize that future research should prioritize rigorous, longitudinal, and biomarker-informed studies to clarify the therapeutic potential of oxytocin in BPD. Several ongoing or recently registered trials are addressing these gaps. For instance, a trial at the University of Freiburg (NCT05285383) is examining the combined effects of oxytocin and psychotherapeutic training on emotion regulation in individuals with personality disorders. Another planned study in Israel (NCT05465753) is investigating oxytocin-enhanced psychotherapy in female patients with trauma histories and interpersonal dysfunction. These trials reflect a growing interest in developing integrative treatment models that tailor oxytocin use to specific therapeutic contexts and patient characteristics. Randomized controlled trials with larger and more diverse samples, including male and gender-diverse participants, are needed to address the current demographic bias and determine differential treatment responsiveness. Furthermore, investigations should explore optimal dosing schedules, timing relative to therapy sessions, and the potential cumulative effects of long-term administration. Integrating genetic screening—such as oxytocin receptor gene (OXTR) polymorphisms—and assessments of early trauma or attachment styles may help identify patient subgroups most likely to benefit from oxytocin-enhanced interventions. Neuroimaging techniques (e.g., fMRI, PET) and real-time monitoring of oxytocin levels could offer mechanistic insights and improve the individualization of treatment. Additionally, combining oxytocin administration with structured therapeutic protocols (e.g., DBT, MBT, EMDR) in manualized, staged approaches may help mitigate risk while maximizing engagement and affective modulation. Finally, ethical considerations, such as the potential for the misuse of or overreliance on pharmacological adjuncts, must be carefully addressed as the field moves toward personalized and integrative treatment models for individuals with BPD.

## 5. Conclusions

In this review, we synthesize the current evidence on intranasal oxytocin as a treatment option for BPD, focusing on the neurobiological mechanisms, emotional and social processing, and clinical outcomes. The evidence indicates that oxytocin modulates the key neural circuits implicated in BPD—particularly amygdala hyperreactivity and prefrontal–limbic connectivity—and may improve emotion regulation and interpersonal sensitivity in certain individuals. However, the clinical effects of oxytocin remain mixed, with the outcomes influenced by factors such as trauma history, sex, and genetic predispositions.

This review also highlights methodological inconsistencies and limitations in the current literature, including small samples, gender imbalance, and limited long-term data. Despite these challenges, the findings support the cautious exploration of oxytocin as an adjunct to psychotherapeutic interventions rather than as a stand-alone treatment. Future research should focus on larger, stratified trials, biomarker-informed participant selection, and integration with established treatment models.

By clearly delineating the current state of evidence, this review provides a grounded foundation for future investigation and clinical innovation in the targeted use of oxytocin for individuals with BPD.

## Data Availability

No new data were created or analyzed in this study.

## Abbreviations

BPD	Borderline Personality Disorder
DBT	Dialectical Behaviour Therapy
MBT	Mentalization-Based Therapy
HPA	Hypothalamic–Pituitary–Adrenal

## Appendix A

**Table A1:** Summary of included studies on oxytocin and borderline personality disorder.

Study	Design	Sample Size	Oxytocin Admin	Outcomes Measured	Key Findings
Bertsch et al. (2013)	RCT	N = 40	Intranasal	Social threat sensitivity.	Reduced social threat, improved
				emotion regulation	regulation
Domes et al. (2007)	RCT	N = 30	Intranasal	Amygdala reactivity	Reduced amygdala activity
Brine et al. (2013)	RCT	N = 34	Intranasal	Interpersonal trust.	improved prosocial behavior
				cooperation	
Simeon et al. (2011)	Plot Study	N = 10	Intranasal	Stress reactivity	Attenuated stress response
Ebert et al. (2013)	Observational	N = 31	Endogenous	interpersonal trust, childhood	Trust modulation moderated by
				trauma	trauma
Lischke et al. (2017)	MRI	N = 28	Intranasal	Amygdala reactivity to	Differential limbic responses in BPD
				emotional scenes	vs controls
Schneider et al. (2020)	RCT	N = 60	Intranasal	Approach-avoidance behavior	Normalized behavioral responses
Domes et al. (2019)	RCT	N = 45	Intranasal	Empathy, approach	Enhanced empathy and social
				motivation	motivation
Macz et al. (2024)	RCT	N = 70	Intranasal	Depressive symptoms.	Improved symptoms in MDD, not BPD
				symptom severity	
Bertsch et al. (2013)	RCT	N = 40	Intranasal	Social threat sensitivity.	Reduced social threat, improved
				emotion regulation	regulation
Domes et al. (2007)	RCT	N = 30	Intranasal	Amygdala reactivity	Reduced amygdala activity
Brine et al. (2013)	RCT	N = 34	Intranasal	Interpersonal trust.	Improved prosocial behavior
				cooperation	
Simeon et al. (2011)	Plot Study	N = 10	Intranasal	Stress reactivity	Attenuated stress response
Ebert et al. (2013)	Observational	N = 31	Endogenous	Interpersonal trust, childhood	Trust modulation moderated by
				trauma	trauma
Lischke et al. (2017)	MRI	N = 28	Intranasal	Amygdala reactivity to	Differential limbic responses in BPD
				emotional scenes	vs controls
Schneider et al. (2020)	RCT	N = 60	Intranasal	Approach-avoidance behavior	Normalized behavioral responses
Domes et al. (2019)	RCT	N = 45	Intranasal	Empathy, approach	Enhanced empathy and social
Brine et al. (2013)	RCT	N = 34	Intranasal	interpersonal trust.	Improved prosocial behavior
				cooperation	
Simeon et al. (2011)	Plot Study	N = 10	Intranasal	Stress reactivity	Attenuated stress response
Ebert et al. (2013)	Conventional	N = 31	Endogenous	Interpersonal trust, childhood	Trust modulation moderated by
				trauma	trauma
Lischke et al. (2017)	fMRI	N = 28	Intranasal	Amygdala reactivity to	Differential limbic responses in BPD
				emotional scenes	vs controls
Schneider et al. (2020)	RCT	N = 60	Intranasal	Approach-avoidance behavior	Normalized behavioral responses
Domes et al. (2019)	RCT	N = 45	Intranasal	Empathy, approach	Enhanced empathy and social
				motivation	motivation
Macz et al. (2024)	RCT	N = 70	Intranasal	Depressive symptoms,	Improved symptoms in MDO, not BPD
				symptom severity	
Bertsch et al. (2013)	RCT	N = 40	Intranasal	Social threat sensitivity	Reduced social threat, improved
				emotion regulation	regulation
Domes et al. (2007)	RCT	N = 30	Intranasal	Amygdala reactivity	Reduced amygdala activity
Brine et al. (2013)	RCT	N = 34	Intranasal	interpersonal trust,	improved prosocial behavior
				cooperation	

**Table A2:** PRISMA 2020 checklist.

Section	Item	Checklist Item	Location in Manuscript
**TITLE**	1	Identify the report as a systematic review.	Title page, Abstract
**ABSTRACT**	2	Structured summary of background, objectives, methods, results, limitations.	Abstract
**INTRODUCTION**	3	Rationale for the review.	Section 1, p. 2
	4	Objectives or research questions addressed.	Section 1, end of p. 2
**METHODS**	5	Eligibility criteria (inclusion/exclusion).	Section 2.2
	6	Information sources (databases, search dates).	Section 2.1
	7	Search strategy, including Boolean terms.	Section 2.1
	8	Selection process (reviewers, consensus).	Section 2.3
	9	Data collection process (who, how, tools).	Section 2.4
	10	Data items (what was extracted).	Section 2.4
	11	Risk of bias assessment methods.	Section 2.5
	12	Effect measures.	Not applicable (narrative synthesis)
	13	Synthesis methods (e.g., rationale for no meta-analysis).	End of Section 2.1 and Section 2.4
	14	Reporting bias assessment.	Not applicable (no meta-analysis)
	15	Certainty of evidence.	Not conducted explicitly (limitations discussed)
**RESULTS**	16	Study selection (numbers, reasons, PRISMA flow).	Section 3.1 + Figure 1
	17	Study characteristics.	Table A1
	18	Risk of bias in studies.	Appendix A
	19	Results of individual studies.	Section 3 (summarized narratively)
	20	Results of syntheses.	Section 3
	21	Reporting biases.	Not applicable
	22	Certainty of evidence.	Not conducted
**DISCUSSION**	23	Summary, limitations, conclusions.	Section 4
**OTHER INFORMATION**	24	Registration and protocol.	Not registered (add if applicable)
	25	Support/funding.	Section 5
	26	Competing interests.	5
	27	Availability of data/code.	Not applicable

## References

[1] American Psychiatric Association (2013). Diagnostic and Statistical Manual of Mental Disorders.

[2] Leichsenring F., Fonagy P., Heim N., Kernberg O.F., Leweke F., Luyten P., Salzer S., Spitzer C., Steinert C. (2024). Borderline personality disorder: A comprehensive review of diagnosis and clinical presentation, etiology, treatment, and current controversies. World Psychiatry.

[3] Zanarini M.C., Frankenburg F.R., Reich D.B., Silk K.R., Hudson J.I., McSweeney L.B. (2007). The subsyndromal phenomenology of borderline personality disorder: A 10-year follow-up study. Am. J. Psychiatry.

[4] Thadani B., Pérez-García A.M., Bermúdez J. (2022). Functional impairment in borderline personality disorder: The mediating role of perceived social support. Front. Psychol..

[5] Sansone R.A., Sansone L.A. (2011). Gender patterns in borderline personality disorder. Innov. Clin. Neurosci..

[6] Sher L., Rutter S., New A., Siever L., Hazlett E. (2018). Gender differences and similarities in aggression, suicidal behavior, and psychiatric comorbidity in borderline personality disorder. Acta Psychiatr. Scand..

[7] Bozzatello P., Blua C., Brandellero D., Baldassarri L., Brasso C., Rocca P., Bellino S. (2024). Gender differences in borderline personality disorder: A narrative review. Front. Psychiatry.

[8] Gunderson J.G., Stout R.L., McGlashan T.H., Shea M.T., Morey L.C., Grilo C.M., Zanarini M.C., Yen S., Markowitz J.C., Sanislow C. (2011). Ten-Year Course of Borderline Personality Disorder: Psychopathology and Function From the Collaborative Longitudinal Personality Disorders Study. Arch. Gen. Psychiatry.

[9] Zanarini M.C., Horwood J., Wolke D., Waylen A., Fitzmaurice G., Grant B.F. (2011). Prevalence of DSM-IV borderline personality disorder in two community samples: 6330 English 11-year-olds and 34,653 American adults. J. Pers. Disord..

[10] Linehan M.M. (1993). Cognitive-Behavioral Treatment of Borderline Personality Disorder.

[11] Bateman A.W., Fonagy P. (2004). Psychotherapy for BPD: Mentalization-Based Treatment.

[12] Chen F., Kumsta R., Dvorak F., Domes G., Yim O.S., Ebstein R.P., Heinrichs M. (2015). Genetic modulation of oxytocin sensitivity: A pharmacogenetic approach. Transl. Psychiatry.

[13] Ebert A., Kolb M., Heller J., Edel M.A., Roser P., Brüne M. (2013). Modulation of interpersonal trust in borderline personality disorder by intranasal oxytocin and childhood trauma. Soc. Neurosci..

[14] Kenkel W.M., Perkeybile A.M., Yee J.R., Pournajafi-Nazarloo H., Lillard T.S., Ferguson E.F., Wroblewski K.L., Ferris C.F., Carter C.S., Connelly J.J. (2019). Behavioral and epigenetic consequences of oxytocin treatment at birth. Sci. Adv..

[15] Kroll F., Powell G.T., Ghosh M., Gestri G., Antinucci P., Hearn T.J., Tunbak H., Lim S., Dennis H.W., Fernandez J.M. (2021). A simple and effective F0 knockout method for rapid screening of behaviour and other complex phenotypes. Elife.

[16] Byrd A.L., Tung I., Manuck S.D., Vine V., Horner M., Hipwell A.E., Stepp S.D. (2021). An interaction between early threat exposure and the oxytocin receptor in females: Disorder-specific versus general risk for psychopathology and social-emotional mediators. Dev. Psychopathol..

[17] Quintana D.S., Lischke A., Grace S., Scheele D., Ma Y., Becker B. (2021). Advances in the field of intranasal oxytocin research: Lessons learned and future directions for clinical research. Mol. Psychiatry.

[18] Meyer-Lindenberg A., Domes G., Kirsch P., Heinrichs M. (2011). Oxytocin and vasopressin in the human brain: Social neuropeptides for translational medicine. Nat. Rev. Neurosci..

[19] Page M.J., McKenzie J.E., Bossuyt P.M., Boutron I., Hoffmann T.C., Mulrow C.D., Shamseer L., Tetzlaff J.M., Akl E.A., Brennan S.E. (2021). The PRISMA 2020 statement: An updated guideline for reporting systematic reviews. BMJ.

[20] Olff M., Frijling J.L., Kubzansky L.D., Bradley B., Ellenbogen M.A., Cardoso C., Bartz J.A., Yee J.R., van Zuiden M. (2013). The role of oxytocin in social bonding, stress regulation, and mental health: An update on the moderating effects of context and interindividual differences. Psychoneuroendocrinology.

[21] Perrotta G. (2020). Oxytocin and the role of “regulator of emotions”: Definition, neurobiochemical and clinical contexts, practical applications, and contraindications. Arch. Depress. Anxiety.

[22] Triana-Del Rio R., Ranade S., Guardado J., LeDoux J., Klann E., Shrestha P. (2022). The modulation of emotional and social behaviors by oxytocin signaling in the limbic network. Front. Mol. Neurosci..

[23] di Giacomo E., Andreini E., Santambrogio J., Arcara A., Clerici M. (2024). The interplay between borderline personality disorder and oxytocin: A systematic narrative review on possible contribution and treatment options. Front. Psychiatry.

[24] Folorunsho I.L., Harry N.M., Udegbe D.C., Jessa D. (2024). Impact of oxytocin on social bonding and its potential as a treatment for social anxiety disorder. World J. Biol. Pharm. Health Sci..

[25] Hilton W. (2024). Oxytocin: Hormone driving social bonding, reproduction, and stress reduction. Autacoids J..

[26] Carpenter R.W., Trull T.J. (2013). Components of emotion dysregulation in borderline personality disorder: A review. Curr. Psychiatry Rep..

[27] Ruocco E., Fusco P., Musone V. (2023). An efficient artificial neural network algorithm for solving boundary integral equations in elasticity. Eng. Anal. Bound. Elem..

[28] Domes G., Heinrichs M., Glascher J., Buchel C., Braus D.F., Herpertz S.C. (2007). Oxytocin attenuates amygdala responses to emotional faces regardless of valence. Biol. Psychiatry.

[29] Domes G., Heinrichs M., Kumbier E., Grossmann A., Hauenstein K., Herpertz S.C. (2013). Effects of intranasal oxytocin on the neural basis of face processing in autism spectrum disorder. Biol. Psychiatry.

[30] Donegan N.H., Sanislow C.A., Blumberg H.P., Fulbright R.K., Lacadie C., Skudlarski P., Gore J.C., Olson I.R., McGlashan T.H., Wexler B.E. (2003). Amygdala hyperreactivity in borderline personality disorder: Implications for emotional dysregulation. Biol. Psychiatry.

[31] Cullen K.R., Vizueta N., Thomas K.M., Han G.J., Lim K.O., Camchong J., Mueller B.A., Bell C.H., Heller M.D., Schulz S.C. (2011). Amygdala functional connectivity in young women with borderline personality disorder. Brain Connect..

[32] Pier K.S., Marin L.K., Wilsnack J., Goodman M. (2016). The neurobiology of borderline personality disorder. Psychiatr. Times.

[33] Geurts D.E.M., Van den Heuvel T.J., Huys Q.J.M., Verkes R.J., Cools R. (2022). Amygdala response predicts clinical symptom reduction in patients with borderline personality disorder: A pilot fMRI study. Front. Behav. Neurosci..

[34] Kirsch P., Esslinger C., Chen Q., Mier D., Lis S., Siddhanti S., Meyer-Lindenberg A. (2005). Oxytocin modulates neural circuitry for social cognition and fear in humans. J. Neurosci..

[35] Bertsch K., Gamer M., Schmidt B., Herpertz S.C. (2013). Oxytocin and reduction of social threat hypersensitivity in women with BPD. Am. J. Psychiatry.

[36] Herpertz S.C., Bertsch K. (2015). A new perspective on the pathophysiology of borderline personality disorder: A model of the role of oxytocin. Am. J. Psychiatry.

[37] Jeung-Maarse H., Schmitgen M.M., Schmitt R. (2023). Oxytocin effects on amygdala reactivity to angry faces in males and females with antisocial personality disorder. Neuropsychopharmacology.

[38] Domes G., Lischke A., Berger C., Grossmann A., Hauenstein K., Heinrichs M., Herpertz S.C. (2010). Effects of intranasal oxytocin on emotional face processing in women. Psychoneuroendocrinology.

[39] New A., Hazlett E., Buchsbaum M. (2007). Amygdala–Prefrontal disconnection in borderline personality disorder. Neuropsychopharmacology.

[40] Lischke A., Domin M., Freyberger H.J., Grabe H.J., Mentel R., Bernheim D., Lotze M. (2015). Structural alterations in white-matter tracts connecting (para-)limbic and prefrontal brain regions in borderline personality disorder. Psychol. Med..

[41] Lischke A., Herpertz S.C., Berger C., Domes G., Gamer M. (2017). Divergent effects of oxytocin on (para-)limbic reactivity to emotional and neutral scenes in females with and without borderline personality disorder. Soc. Cogn. Affect. Neurosci..

[42] Lei X., Zhong M., Zhang B., Yang H., Peng W., Liu Q., Zhang Y., Yao S., Tan C., Yi J. (2019). Structural and functional connectivity of the anterior cingulate cortex in patients with borderline personality disorder. Front. Neurosci..

[43] Minzenberg M., Laird A., Thelen S., Carter C., Glahn D. (2009). Meta-analysis of 41 Functional Neuroimaging Studies of Executive Function in Schizophrenia. Arch. Gen. Psychiatry.

[44] Preißler S., Dziobek I., Ritter K., Heekeren H.R., Roepke S. (2010). Social Cognition in Borderline Personality Disorder: Evidence for Disturbed Recognition of the Emotions, Thoughts, and Intentions of others. Front. Behav. Neurosci..

[45] Dziobek I., Preißler S., Grozdanovic Z., Heuser I., Heekeren H.R., Roepke S. (2011). Neuronal correlates of altered empathy and social cognition in borderline personality disorder. Neuroimage.

[46] Sharp C., Pane H., Ha C., Venta A., Patel A.B., Sturek J., Fonagy P. (2011). Theory of mind and emotion regulation difficulties in adolescents with borderline traits. J. Am. Acad. Child Adolesc. Psychiatry.

[47] Roepke S., Vater A., Preißler S., Heekeren H.R., Dziobek I. (2013). Social cognition in borderline personality disorder. Front. Neurosci..

[48] Galvez-Merlin A., Lopez-Villatoro J.M., de la Higuera-Gonzalez P., de la Torre-Luque A., Reneses-Prieto B., Diaz-Marsa M., Carrasco J.L. (2024). Social cognition deficits in borderline personality disorder: Clinical relevance. Psychiatry Res..

[49] Hurlemann R., Patin A., Onur O.A., Cohen M.X., Baumgartner T., Metzler S., Kendrick K.M. (2010). Oxytocin enhances amygdala-dependent, socially reinforced learning and emotional empathy in humans. J. Neurosci..

[50] Bartz J.A., Zaki J., Bolger N., Ochsner K.N. (2011). Social effects of oxytocin in humans: Context and person matter. Trends Cogn. Sci..

[51] Kis A., Kemerle K., Hernádi A., Topál J. (2013). Oxytocin and social pretreatment have similar effects on processing of negative emotional faces in healthy adult males. Front. Psychol..

[52] Ebert A., Edel M.A., Gilbert P., Brüne M. (2018). Endogenous oxytocin is associated with the experience of compassion and recalled upbringing in Borderline Personality Disorder. Depress. Anxiety.

[53] Tillman R., Gordon I., Naples A., Rolison M., Leckman J.F., Feldman R., Pelphrey K.A., McPartland J.C. (2019). Oxytocin enhances the neural efficiency of social perception. Front. Hum. Neurosci..

[54] Korisky A., Gordon I., Goldstein A. (2022). Oxytocin impacts top-down and bottom-up social perception in adolescents with ASD: A MEG study of neural connectivity. Mol. Autism.

[55] Foxhall M., Hamilton-Giachritsis C., Button K. (2019). The link between rejection sensitivity and borderline personality disorder: A systematic review and meta-analysis. Br. J. Clin. Psychol..

[56] Sato M., Fonagy P., Luyten P. (2020). Rejection sensitivity and BPDfeatures: The mediating roles of attachment anxiety, need to belong, and self-criticism. J. Personal. Disord..

[57] Brüne M., Ebert A., Kolb M., Tas C., Edel M.A., Roser P. (2013). Oxytocin influences avoidant reactions to social threat in adults with borderline personality disorder. Hum. Psychopharmacol..

[58] Bertsch K., Herpertz S.C. (2018). Oxytocin and borderline personality disorder. Curr. Top. Behav. Neurosci..

[59] Schneider I., Boll S., Volman I., Roelofs K., Spohn A., Herpertz S.C., Bertsch K. (2020). Oxytocin normalizes approach–avoidance behavior in women with borderline personality disorder. Front. Psychiatry.

[60] Domes G., Ower N., von Dawans B., Spengler F.B., Dziobek I., Bohus M., Matthies S., Philipsen A., Heinrichs M. (2019). Effects of intranasal oxytocin administration on empathy and approach motivation in women with borderline Kullakpersonality disorder: A randomized controlled trial. Transl. Psychiatry.

[61] Jawad M.Y., Ahmad B., Hashmi A.M. (2021). Role of oxytocin in the pathogenesis and modulation of borderline personality disorder: A review. Cureus.

[62] Mielke E.L., Koenig J., Herpertz S.C., Steinmann S., Neukel C., Kilavuz P., van der Venne P., Bertsch K., Kaess M. (2023). Adverse childhood experiences mediate the negative association between BPDsymptoms and plasma oxytocin. Prog. Neuro-Psychopharmacol. Biol. Psychiatry.

[63] Carrasco J.L., Díaz-Marsá M., Pastrana J.I., Molina R., Brotons L., López-Ibor M.I., López-Ibor J.J. (2007). Hypothalamic-pituitary-adrenal axis response in borderline personality disorder without post-traumatic features. Br. J. Psychiatry.

[64] Jobst A., Padberg F., Mauer M.C., Daltrozzo T., Bauriedl-Schmidt C., Sabass L., Sarubin N., Falkai P., Renneberg B., Zill P. (2016). Lower Oxytocin Plasma Levels in Borderline Patients with Unresolved Attachment Representations. Front. Hum. Neurosci..

[65] Thomas N., Gurvich C., Hudaib A.R., Gavrilidis E., Kulkarni J. (2019). Systematic review and meta-analysis of basal cortisol levels in borderline personality disorder compared to non-psychiatric controls. Psychoneuroendocrinology.

[66] Kulakova E., Graumann L., Wingenfeld K. (2024). The hypothalamus-pituitary-adrenal axis and social cognition in borderline personality disorder. Curr. Neuropharmacol..

[67] Bourvis N., Aouidad A., Cabelguen C., Cohen D., Xavier J. (2017). How do stress exposure and stress regulation relate to borderline personality disorder?. Front. Psychol..

[68] Franczak Ł., Podwalski P., Wysocki P., Dawidowski B., Jędrzejewski A., Jabłoński M., Samochowiec J. (2024). Impulsivity in ADHD and borderline personality disorder: A systematic review of gray and white matter variations. J. Clin. Med..

[69] Ito E., Shima R., Yoshioka T. (2019). A novel role of oxytocin: Oxytocin-induced well-being in humans. Biophys. Physicobiology.

[70] Yeğen B.Ç. (2010). Oxytocin and hypothalamo-pituitary-adrenal axis. Marmara Pharm. J..

[71] Li Y., Hassett A.L., Seng J.S. (2019). Exploring the mutual regulation between oxytocin and cortisol as a marker of resilience. Arch. Psychiatr. Nurs..

[72] Karin O., Raz M., Tendler A., Bar A., Korem Kohanim Y., Milo T., Alon U. (2020). A new model for the HPA axis explains dysregulation of stress hormones on the timescale of weeks. Mol. Syst. Biol..

[73] Young Kuchenbecker S., Pressman S.D., Celniker J., Grewen K.M., Sumida K.D., Jonathan N., Everett B., Slavich G.M. (2021). Oxytocin, cortisol, and cognitive control during acute and naturalistic stress. Stress.

[74] Uvnäs-Moberg K., Gross M.M., Calleja-Agius J., Turner J.D. (2024). The yin and yang of the oxytocin and stress systems: Opposites, yet interdependent and intertwined determinants of lifelong health trajectories. Front. Endocrinol..

[75] Simeon D., Bartz J., Hamilton H., Crystal S., Braun A., Ketay S., Hollander E. (2011). Oxytocin administration attenuates stress reactivity in borderline personality disorder: A pilot study. Psychoneuroendocrinology.

[76] Uzar M., Dmitrzak-Węglarz M., Słopień A. (2024). The Role of Oxytocin and Vasopressin in People with Borderline Personality Disorder: A Closer Look at Adolescents. Int. J. Mol. Sci..

[77] Liu J., Kou J., Tan L., Li H., Lei Y. (2025). The complex role of oxytocin in fear acquisition and generalization. Psychoneuroendocrinology.

[78] Takayanagi Y., Onaka T. (2021). Roles of oxytocin in stress responses, allostasis, and resilience. Int. J. Mol. Sci..

[79] Lin Y.T., Chen C.C., Huang C.C., Nishimori K., Hsu K.-S. (2017). Oxytocin stimulates hippocampal neurogenesis via oxytocin receptor expressed in CA3 pyramidal neurons. Nat. Commun..

[80] Pekarek B.T., Hunt P.J., Arenkiel B.R. (2020). Oxytocin and sensory network plasticity. Front. Neurosci..

[81] Harvey A.R. (2020). Links between the neurobiology of oxytocin and human musicality. Front. Hum. Neurosci..

[82] Froemke R.C., Young L.J. (2021). Oxytocin, neural plasticity, and social behavior. Annu. Rev. Neurosci..

[83] Onaka T., Takayanagi Y. (2021). The oxytocin system and early-life experience-dependent plastic changes. J. Neuroendocrinol..

[84] Bethlehem R.A.I., Baron-Cohen S., van Honk J., Auyeung B., Allison C. (2013). The oxytocin paradox. Front. Behav. Neurosci..

[85] Gur R., Tendler A., Wagner S. (2014). Long-term social recognition memory is mediated by oxytocin-dependent synaptic plasticity in the medial amygdala. Biol. Psychiatry.

[86] Lee S.Y., Park S.H., Chung C., Kim J.J., Choi S.-Y., Han J.-S. (2015). Oxytocin protects hippocampal memory and plasticity from uncontrollable stress. Sci. Rep..

[87] Bukatova S., Reichova A., Bacova Z., Bakos J. (2023). Neonatal oxytocin treatment alters levels of precursor and mature BDNF forms and modifies the expression of neuronal markers in the male rat hippocampus. Neuropeptides.

[88] Khazen T., Narattil N.R., Ferreira G., Maroun M. (2023). Hippocampal oxytocin is involved in spatial memory and synaptic plasticity deficits following acute high-fat diet intake in juvenile rats. Cereb. Cortex.

[89] Chavez J., Le A.A., Lauterborn J.C., Cox B.M., Jia Y., Lynch G., Gall C.M. (2024). Early-life oxytocin rescues hippocampal synaptic plasticity and episodic memory in a mouse model of Fragile X syndrome. bioRxiv..

[90] Alaerts K., Bernaerts S.P. (2019). 509 Continual oxytocin treatment induces long-lasting adaptations within amygdala circuitry in autism: A randomized placebo-controlled trial. Eur. Neuropsychopharmacol..

[91] Alaerts K., Bernaerts S., Prinsen J., Dillen C., Steyaert J., Wenderoth N. (2020). Oxytocin induces long-lasting adaptations within amygdala circuitry in autism: A treatment-mechanism study with randomized placebo-controlled design. Neuropsychopharmacology.

[92] Zagorski N. (2020). Oxytocin may promote empathy in BPD patients. Psychiatr. News.

[93] Gao F. (2023). The function of oxytocin in memory: A general review of oxytocin’s effect on memory. World J. Neurosci..

[94] Maoz H., Grossman-Giron A., Sedoff O., Nitzan U., Kashua H., Yarmishin M., Arad O., Tzur Bitan D. (2024). Intranasal oxytocin as an adjunct treatment among patients with severe major depression with and without comorbid borderline personality disorder. J. Affect. Disord..

[95] Mercedes Perez-Rodriguez M., Mahon K., Russo M., Ungar A.K., Burdick K.E. (2015). Oxytocin and social cognition in affective and psychotic disorders. Eur. Neuropsychopharmacol..

[96] Bradley E.R., Woolley J.D. (2017). Oxytocin effects in schizophrenia: Reconciling mixed findings and moving forward. Neurosci. Biobehav. Rev..

[97] Kartal F., Uğur K., Mete B., Demirkol M.E., Tamam L. (2022). The relationship between the oxytocin level and rejection sensitivity, childhood traumas, and attachment styles in borderline personality disorder. Psychiatry Investig..

[98] Bernaerts S., Boets B., Steyaert J., Wenderoth N., Alaerts K. (2020). Oxytocin treatment attenuates amygdala activity in autism: A treatment-mechanism study with long-term follow-up. Transl. Psychiatry.

[99] Miller G., Chen E., Cole S.W. (2019). Health psychology: Developing biologically plausible models linking the social world and physical health. Annu. Rev. Psychol..

[100] Monks D.T., Palanisamy A. (2021). Oxytocin: At birth and beyond. A systematic review of the long-term effects of peripartum oxytocin. Anaesthesia.

[101] Krol K.M., Namaky N., Monakhov M.V., Lai P.S., Ebstein R., Grossmann T. (2021). Genetic variation in the oxytocin system and its link to social motivation in human infants. Psychoneuroendocrinology.

[102] Mellentin A.I., Wallhed Finn S., Skøt L., Thaysen-Petersen D., Mistarz N., Fink-Jensen A., Grüner Nielsen D. (2023). The effectiveness of oxytocin for treating substance use disorders: A systematic review of randomized placebo-controlled trials. Neurosci. Biobehav. Rev..

[103] Ellenbogen M.A., Cardoso C., Serravalle L., Vadaga K., Joober R. (2024). The effects of intranasal oxytocin on the efficacy of psychotherapy for major depressive disorder: A pilot randomized controlled trial. Psychol. Med..

[104] Zhang L., Qu Y., Li L., Sun Y., Qian W., Xiao J., Huang K., Han X., Niu H., Li L. (2025). PVN–NAc Shell–VP Circuit OT and OTR Neurons Regulate Pair Bonding via D2R and D1R. J. Neurosci..

[105] Servan A., Brunelin J., Poulet E. (2018). The effects of oxytocin on social cognition in borderline personality disorder. L’Encéphale.

[106] Teicher M.H., Samson J.A., Anderson C.M., Ohashi K. (2016). The effects of childhood maltreatment on brain structure, function, and connectivity. Nat. Rev. Neurosci..

[107] Zashchirinskaia O., Isagulova E. (2023). Childhood trauma as a risk factor for high-risk behaviors in adolescents with borderline personality disorder. Iran. J. Psychiatry.

[108] Jiang B. (2024). Prediction of BPD based on childhood trauma with the mediating role of experiential avoidance. Front. Psychiatry.

